# A dysphagia model with denervation of the pharyngeal constrictor muscles in guinea pigs: functional evaluation of swallowing

**DOI:** 10.3389/fneur.2024.1401982

**Published:** 2024-06-19

**Authors:** Keiko Hashimoto, Yoichiro Sugiyama, Mami Kaneko, Shota Kinoshita, Ryota Yamamoto, Tomoya Ishida, Toshiro Umezaki, Shigeru Hirano

**Affiliations:** ^1^Department of Otolaryngology-Head and Neck Surgery, Kyoto Prefectural University of Medicine, Kyoto, Japan; ^2^Department of Otolaryngology-Head and Neck Surgery, Faculty of Medicine, Saga University, Saga, Japan; ^3^Department of Otolaryngology-Head and Neck Surgery, Fukuoka Sanno Hospital, Fukuoka, Japan; ^4^Department of Speech and Hearing Sciences, International University of Health and Welfare, and the Voice and Swallowing Center, Fukuoka Sanno Hospital, Fukuoka, Japan

**Keywords:** dysphagia, swallowing, guinea pig, pharyngeal branch of the vagus nerve, videofluoroscopy

## Abstract

**Introduction:**

Swallowing impairment is a crucial issue that can lead to aspiration, pneumonia, and malnutrition. Animal models are useful to reveal pathophysiology and to facilitate development of new treatments for dysphagia caused by many diseases. The present study aimed to develop a new dysphagia model with reduced pharyngeal constriction during pharyngeal swallowing.

**Methods:**

We analyzed the dynamics of pharyngeal swallowing over time with the pharyngeal branches of the vagus nerve (Ph-X) bilaterally or unilaterally transected, using videofluoroscopic assessment of swallowing in guinea pigs. We also evaluated the detailed anatomy of the pharyngeal constrictor muscles after the denervation.

**Results:**

Videofluoroscopic examination of swallowing showed a significant increase in the pharyngeal area during swallowing after bilateral and unilateral sectioning of the Ph-X. The videofluoroscopy also showed significantly higher pharyngeal transit duration for bilateral and unilateral section groups. The thyropharyngeal muscle on the sectioned side was significantly thinner than that on the intact side. In contrast, the thickness of the cricopharyngeal muscles on the sectioned and intact sides were not significantly different. The mean thickness of the bilateral thyropharyngeal muscles showed a linear correlation to the pharyngeal area and pharyngeal transit duration.

**Discussion:**

Data obtained in this study suggest that denervation of the Ph-X could influence the strength of pharyngeal contraction during pharyngeal swallowing in relation to thickness of the pharyngeal constrictor muscles, resulting in a decrease in bolus speed. This experimental model may provide essential information (1) for the development of treatments for pharyngeal dysphagia and (2) on the mechanisms related to the recovery process, reinnervation, and nerve regeneration following injury and swallowing impairment possibly caused by medullary stroke, neuromuscular disease, or surgical damage from head and neck cancer.

## Introduction

Early and proper assessment of swallowing impairment is crucial in preventing aspiration. However, dysphagia is more likely to occur in the elderly population due to age-related diseases such as cerebrovascular disorders, neurodegenerative diseases, and malignant tumors (1–4). It is a critical issue that can lead to aspiration, pneumonia, and malnutrition. Swallowing is a complex behavior that involves the sequential contraction of many swallowing-related muscles (5, 6). It is controlled by neural pathways called the swallowing central pattern generators (CPG) in the brainstem (6–9). Because of this complexity, it is essential to comprehend the pathophysiology of dysphagia. Spatiotemporal stereotyped movements during the pharyngeal stage of swallowing include laryngeal elevation, pharyngeal constriction, glottal adduction, and cricopharyngeal opening; these movements are driven by the swallowing CPG, and activated by peripheral afferent signals from the pharynx and larynx. The reflexibility of swallowing can also be mediated by descending signals from the higher brain centers, as well as by other afferents in the spinal cord (10–15). In particular, the sequential activation of pharyngeal constrictor muscles (e.g., thyropharyngeal muscle) is critical for efficient bolus transfer during swallowing. Deteriorated movement of these muscles can cause attenuated pharyngeal contraction, possibly resulting in severe pharyngeal dysphagia. These movements involve well-coordinated swallow-breathing coordination to prevent aspiration (16).

Animal models have proven useful in revealing the pathophysiology and to facilitate development of new treatments for dysphagia caused by many diseases, such as amyotrophic lateral sclerosis, Parkinson’s disease, and stroke (17–21). To understand the mechanisms for various patterns of dysphagia and to develop effective treatments for dysphagia, animal models are still necessary to test the causal mechanisms that produce swallowing disorders (22–24). A sensory deficit, which results from inadequate sensory information from the bolus passage into the pharynx to the swallowing CPG, can cause a delayed incidence of swallowing (2, 6, 25–27). To examine swallowing function, the animal model for this sensory deficit has already been developed by transecting the superior laryngeal nerve, a significant afferent pathway to evoke pharyngeal swallowing (28, 29).

However, the most essential factor that renders a patient incapable of swallowing is the reduction of the swallowing-related muscle activity during swallowing, which can result in severe aspiration pneumonia. Thus, assessing swallowing function for dysphagia caused by the impairment of swallow motor activity in relation to larynx excursion and hyoid movement in animal models is crucial (18, 30). King et al. (31) have developed a dysphagia animal model with attenuated jaw excursion produced by injury of the mylohyoid muscle. While the immobility of the pharynx and larynx due to bulbar paralysis, possibly caused by medullary stroke (e.g., Wallenberg syndrome), leads to severe pharyngeal dysphagia, animal models with attenuated pharyngeal contraction during swallowing have not been well developed (32–36).

On the other hand, ethical concerns should be considered when developing an animal model with chronic impaired swallowing function. The animals with severe dysphagia may suffer from an intolerable condition, such as chronic aspiration, likely leading to continuous body weight loss. As such, a suitable animal model representing swallowing motor impairment must be developed without any chronic distress during the course of a long-term study.

The present study was aimed to establish a new dysphagia model with reduced pharyngeal constriction during pharyngeal swallowing. We also purposed development of an animal model of a specific motor deficiency related to pharyngeal swallowing, which does not suffer severe complications, such as chronic aspiration, severe body weight loss, and the lack of oral intake. In guinea pigs, using videofluoroscopic assessment of swallowing, we analyzed the dynamics of pharyngeal swallowing over time with the pharyngeal branches of the vagus nerve (Ph-X) bilaterally or unilaterally transected. We also evaluated the anatomy of the pharyngeal constrictor muscles after the denervation.

## Methods

All experimental procedures, approved by the local Universal Committee for the Use of Animals in Research (M2022-314), were performed on 10 guinea pigs (Hartley, male, Shimizu Laboratory Supplies, Kyoto, Japan) weighing 450–520 g and were confirmed by the Physiological Society of Japan Principles for the Care and Use of Animals.

### Surgery and recording procedures

**Figure 1:**
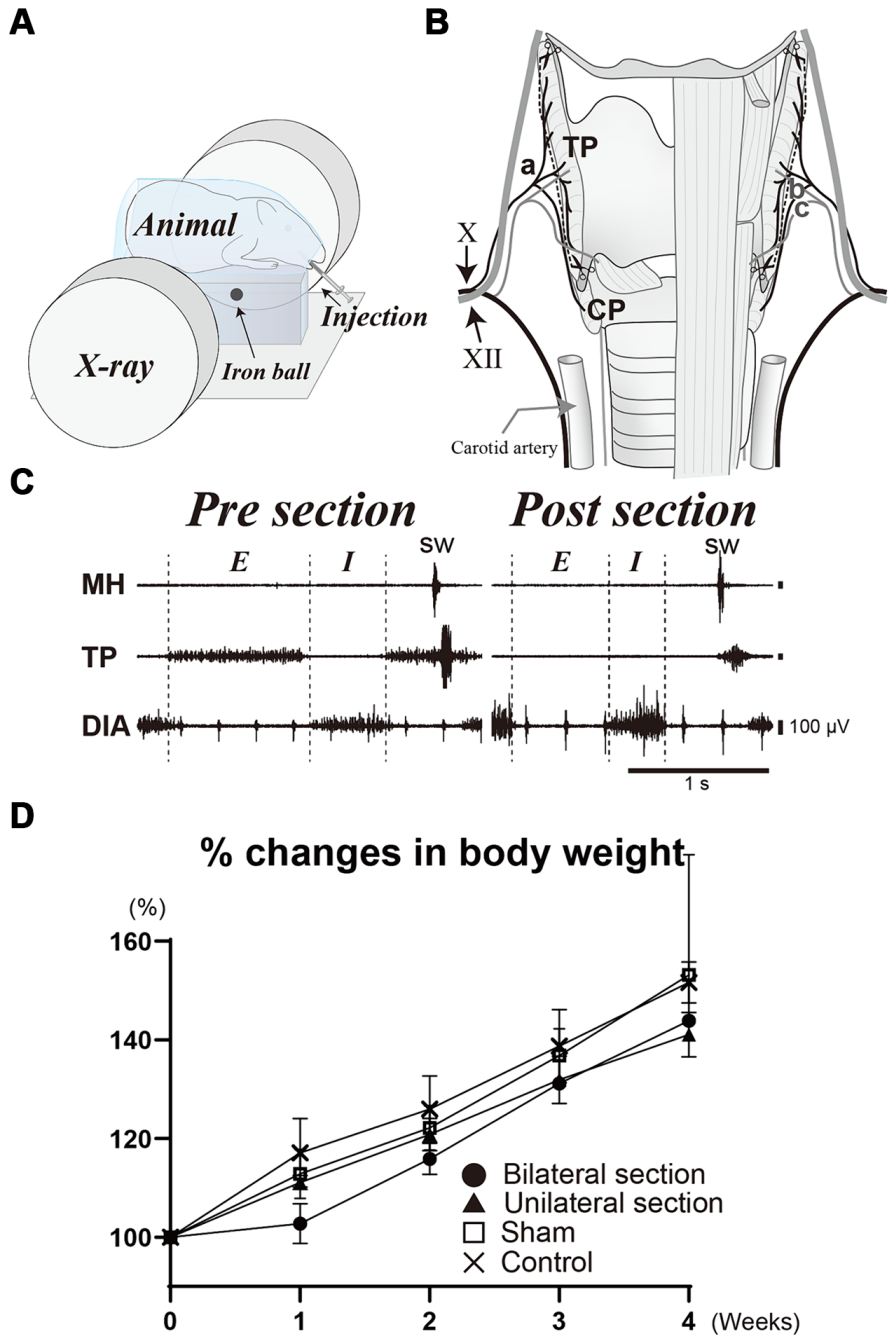
**(A)** Illustration of videofluoroscopic examination of swallowing. **(B)** Schema of surgical procedure for the denervation dysphagia model. Bilateral sectioning of the pharyngeal branch of the vagus nerve (Ph-X) (a) is performed (dashed line) while preserving the internal (b) and external (c) branches of the superior laryngeal nerve. **(C)** A representative recording of activities of the mylohyoid (MH), thyropharyngeal (TP), and diaphragm (DIA) muscles during breathing and swallowing before and after the section of the Ph-X. Body weight changes for bilateral (closed circle), unilateral Ph-X section (closed triangle), sham (open square), and control groups (cross) are shown in panel **(D)**. X, vagus nerve; XII, hypoglossal nerve; E, expiratory phase; I, inspiratory phase; CP, cricopharyngeal muscle; sw, swallowing.

Eight animals were prepared for the surgery groups, whereas the remaining two animals were used as radiographic and histological control groups. Prior to the inclusion of the study, animals were gently handled for several days to be compliant in the recording apparatus of the videofluoroscopy to immobilize the animals for a restraint period of at least 30 min without any indicators of distress. Initially, the videofluoroscopic examination of swallowing was performed in all animals using an X-ray fluoroscopy system (SXT-9000A, Toshiba Medical Manufacturing Co. Ltd., Tochigi, Japan) (44 mV, 0.4 mA, 30 frames/s). The animal was placed in a polyethylene terephthalate cylindrical tube with a small iron ball (10 mm in diameter) to stabilize the animal for subsequent swallowing sessions and to standardize the images obtained from the X-ray system (Figure 1A). Sequential pharyngeal swallowing was induced by the infusion of contrast medium (Iotrolan, 270 mg/mL, viscosity of approximately 8.6 mPa·s, Bayer Yakuhin, Ltd., Osaka, Japan) into the oral cavity using a 1 mL syringe. A series of two or three trials was performed at each time point for all animals tested.

Surgeries were conducted using aseptic procedures in the dedicated operating room, and anesthesia was initiated and maintained with a constant infusion of isoflurane (4% for induction, 1–2.5% for maintenance) following intramuscular injections of dexamethasone (1 mg/kg) and atropine sulfate (0.1 mg/kg) to minimize laryngeal edema and secretion. The level of anesthesia was carefully maintained without any reflexive movement during the surgery, and without a decrease in breathing rate below 20 cycles/min. A midline incision of the cervical skin was made and exposed the larynx. The superior laryngeal nerve was bilaterally identified to avoid mechanical damage during the surgery. The pharyngeal branch of the vagus nerve (Ph-X) was then identified alongside the lateral border of the thyroid cartilage (Figure 1B). We divided all animals into four groups in order to compare the denervation effect on swallowing function. Group 1 (*n* = 3) was animals whose Ph-X was bilaterally cut (bilateral section), while Group 2 (*n* = 3) animals underwent unilateral Ph-X section (unilateral section), Group 3 (*n* = 2) were sham-operated animals, and Group 4 (*n* = 2) were intact animals (control). We used two animals each for sham and control groups, according to the animal reduction approach. For injured animals, three animals were tested to investigate the effects of the nerve denervation, due to the variation in experimental variability within each group. The activities of mylohyoid, thyropharyngeal (TP), and diaphragm muscles were recorded under anesthesia using bipolar hooked stainless steel wire electrodes (insulated except for the tips) during breathing and swallowing; swallowing was evoked by infusion of a small amount of water into the oral cavity. The denervation procedure was deemed to be adequate as respiratory- and swallowing-related activities of the TP muscle were substantially attenuated after the denervation of the Ph-X (Figure 1C). To minimize possible damage caused by the electrodes on the TP muscles, we tested the effects of the section of the Ph-X only in representative animals for each group. After the skin incision was closed by suturing, anesthesia was gradually terminated, and the animals were observed in the recovery cage with a heat lamp for 1 h. Videofluoroscopic examination of swallowing was performed before the surgery and once each week after the surgery. Approximately 1 month after the surgery, animals were sacrificed by using an overdose of pentobarbital administration (intraperitoneally) and were perfused transcardially with 4% paraformaldehyde following physiological saline.

### Histological and data analysis procedures

Consecutive sagittal sections of the larynx and pharynx (10 μm thickness) were made using a freezing microtome and stained with hematoxylin and eosin (Merck KGaA, Darmstadt, Germany) and photographed using a Biorevo BZ-X700 microscope (Keyence, Osaka, Japan). The thickness of the thyropharyngeal (TP) and cricopharyngeal (CP) muscles was measured at four and three different rostrocaudal levels in the sections at approximately 600 μm lateral from the midline, respectively. The body weight of each animal was measured every week.

Muscle activities were amplified and filtered (MEG-5200; Nihon Kohden, Tokyo, Japan) and input into a computer through the analog-digital converter (Power 1401 mk2 data collection system), and the signals were sampled at 5 kHz using Spike2 software (Cambridge Electronic Design, Cambridge, UK).

**Figure 2:**
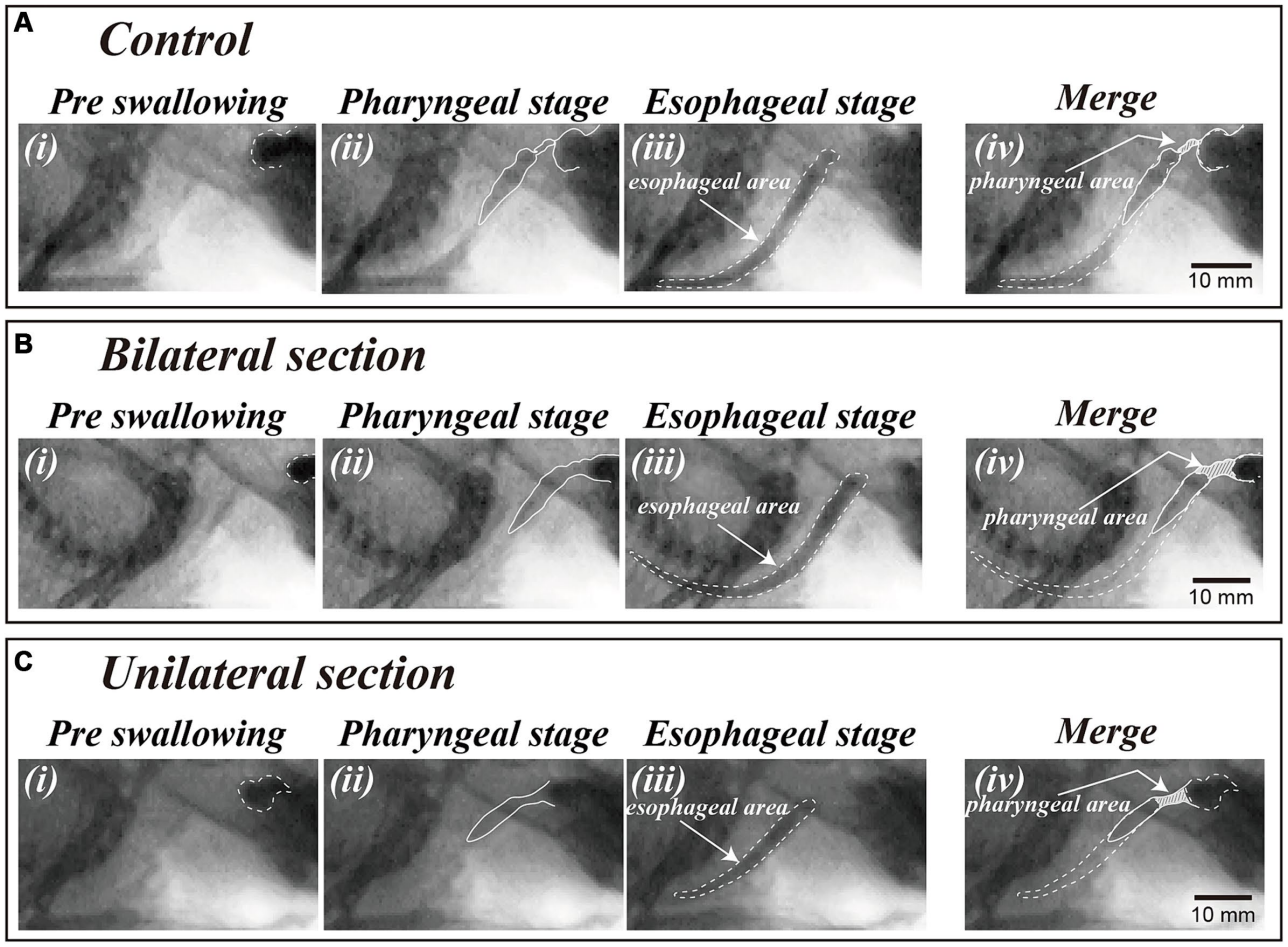
Video-fluoroscopic examination of swallowing in a control animal **(A)** and a dysphagia model 1 month after bilateral **(B)** and unilateral **(C)** sectioning of the Ph-X during oral **(i)**, pharyngeal **(ii)**, and esophageal stages of swallowing **(iii)**. The areas of contrast medium during the oral and esophageal stage of swallowing are depicted by dashed lines in panels **(i,iii)** [i.e., esophageal area in panel **(iii)**]. The dye areas in the pharyngeal cavity during bolus transfer in the pharyngeal stage of swallowing are shown by shaded areas in panel **(iv)** (i.e., pharyngeal area) delineated with reference to the oral **(i)** and esophageal area **(iii)**.

A video editing system (Premiere Pro, Adobe Systems, San Jose, CA, USA) was used to measure the temporal parameters. The dye areas of the contrast solution before swallowing (Figures 2A,Bi), during the pharyngeal stage of swallowing (Figures 2A,Bii), and during the esophageal stage of swallowing (Figures 2A,Biii) were delineated using images extracted from the videos (in three representative swallows). The actual areas were then calculated with reference to the iron ball attached to the recording apparatus. The pharyngeal area at the maximal contraction time of the pharynx during the pharyngeal stage of swallowing was measured [shaded areas in Figures 2A,Biv]. The esophageal area was also measured to evaluate bolus volume for each swallowing. The pharyngeal area was dependent upon the bolus volume and the force of the pharyngeal constriction (37, 38). The pharyngeal transit duration was defined as the period from the initiation of pharyngeal swallowing to the time when the bolus tail passed through the esophageal entrance and was measured in more than three episodes.

Statistical analyses were performed using Prism 10 software (GraphPad Software, San Diego, CA, USA). *t*-Test and single regression analysis were applied to determine the influences of the Ph-X sections on the swallowing-related muscles and swallowing function. Pooled data are presented as means ± standard error. Statistical significance was assumed for *p* < 0.05.

## Results

The present study was aimed to investigate the effects of sectioning the Ph-X on the swallowing function of guinea pigs. All animals tested tolerated well without lethal conditions with the protocols in this study. Consistent increases in body weight were observed in all animals sampled during the course of this study, although a slight decline in their typical trend of weight increase was noted 1 week after the surgery in the bilateral section group (within less than 5%). Aspiration was not observed on videofluoroscopy before or after the surgery, and the percent increases in body weight were not statistically different among all groups tested (One-way ANOVA) (Figure 1D).

**Figure 3:**
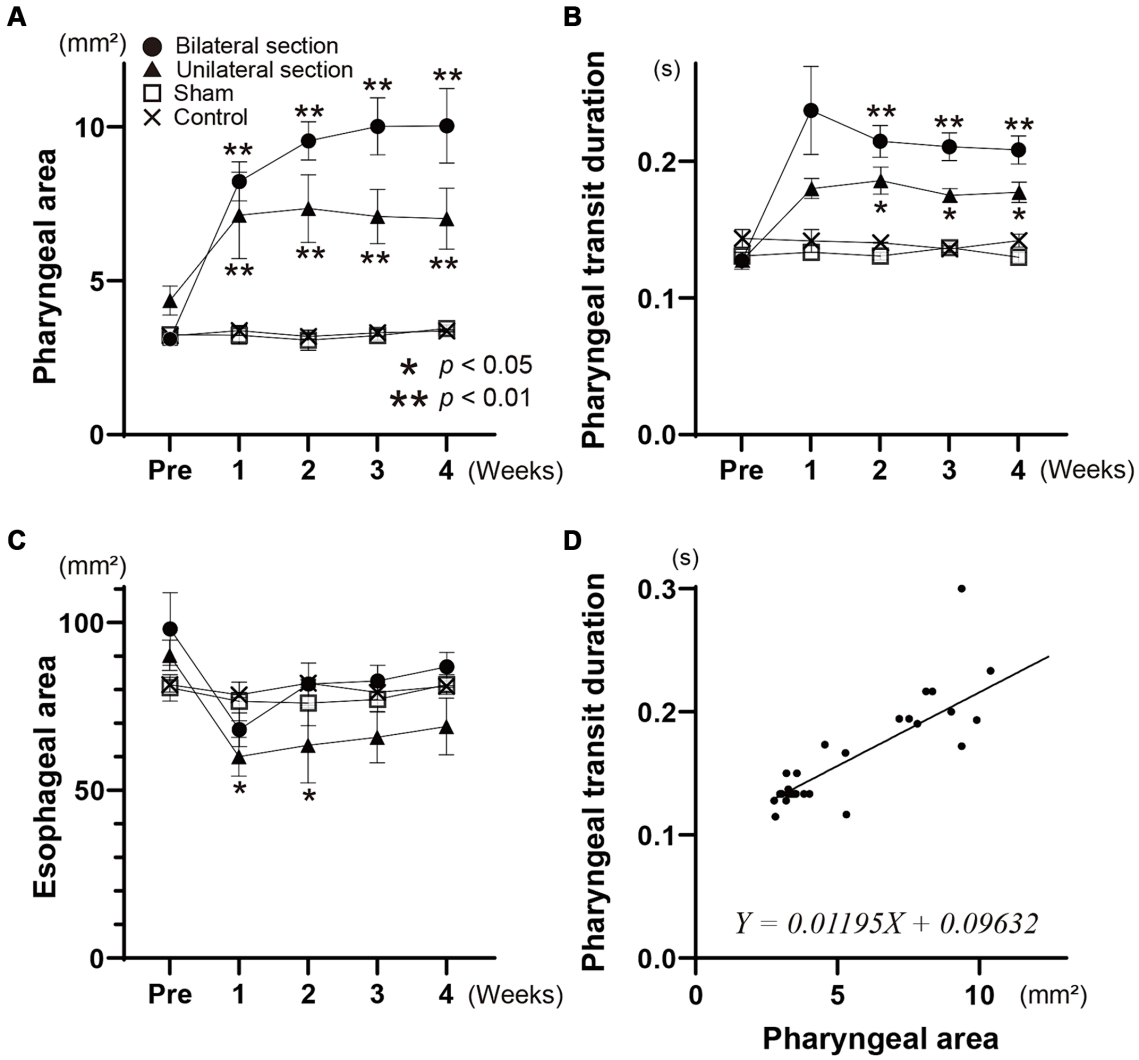
Changes in mean areas of the contrast dye in the pharynx **(A)** and esophagus **(B)** are indicated during the pharyngeal and esophageal stages of swallowing for all groups, respectively. The time course of the pharyngeal transit duration for each group is shown in panel **(C)**. Scatter plots, and relationships between the pharyngeal area and pharyngeal transit duration are represented in panel **(D)**. The formula of the linear regression line is given in panel **(D)**. Different symbols are used to indicate each group in panels **(A–C)**. Closed circle, bilateral section group; closed triangle, unilateral section group; open square, sham-operated group; cross, control group.

Videofluoroscopic examination of swallowing for the bilateral and unilateral section group showed significant increases in the pharyngeal area after the surgery (One-way ANOVA, Tukey’s multiple comparisons test, *p* < 0.01), and the areas for bilateral section group were significantly higher compared with those for unilateral section group (One-way ANOVA, Tukey’s multiple comparisons test, *p* < 0.01) (Figures 2, 3A and Table 1). There was no statistical significance between sham and control groups (One-way ANOVA, Tukey’s multiple comparisons test, *p* = 0.9998) (Figure 3A and Table 1).

**Table 1:** Spatiotemporal and histological analyses regarding swallowing in different experimental groups.

Groups	Videofluoroscopic examination of swallowing						Tissue	
		Pre-surgery	Post-surgery (weeks)				Mean TP thickness (mm)	Mean CP thickness (mm)
			1	2	3	4		
Bilateral section (*n* = 3)	Pharyngeal area (mm^2^)	3.11 ± 0.20	8.22 ± 0.64**	9.55 ± 0.62**	10.0 ± 0.92**	10.0 ± 1.21**	0.55 ± 0.03*	0.64 ± 0.03
	Esophageal area (mm^2^)	98.1 ± 10.8	68.0 ± 4.98	81.6 ± 6.26	82.5 ± 4.64	86.8 ± 4.26		
	Pharyngeal transit duration (s)	0.13 ± 0.01	0.24 ± 0.03	0.21 ± 0.01**	0.21 ± 0.01**	0.21 ± 0.01**		
Unilateral section (*n* = 3)	Pharyngeal area (mm^2^)	4.38 ± 0.47	7.15 ± 1.40**	7.37 ± 1.10**	7.12 ± 0.88**	7.05 ± 0.99**	0.63 ± 0.04	0.64 ± 0.03
	Esophageal area (mm^2^)	90.2 ± 4.51	60.0 ± 5.76*	63.4 ± 11.1*	65.8 ± 7.67	69.0 ± 8.46		
	Pharyngeal transit duration (s)	0.13 ± 0.01	0.18 ± 0.01	0.19 ± 0.01*	0.18 ± 0.00*	0.18 ± 0.01*		
Sham (*n* = 2)	Pharyngeal area (mm^2^)	3.27 ± 0.08	3.26 ± 0.28	3.09 ± 0.32	3.24 ± 0.15	3.47 ± 0.11	0.77 ± 0.00	0.64 ± 0.02
	Esophageal area (mm^2^)	80.5 ± 3.91	76.5 ± 5.73	76.0 ± 6.76	77.0 ± 3.76	81.5 ± 2.88		
	Pharyngeal transit duration (s)	0.13 ± 0.00	0.13 ± 0.00	0.13 ± 0.00	0.14 ± 0.00	0.13 ± 0.00		
Control (*n* = 2)	Pharyngeal area (mm^2^)	3.23 ± 0.03	3.41 ± 0.16	3.21 ± 0.09	3.34 ± 0.11	3.39 ± 0.03	0.79 ± 0.03	0.71 ± 0.00
	Esophageal area (mm^2^)	81.5 ± 2.21	78.5 ± 0.78	82.0 ± 1.16	79.3 ± 2.28	81.1 ± 0.22		
	Pharyngeal transit duration (s)	0.14 ± 0.00	0.14 ± 0.01	0.14 ± 0.00	0.14 ± 0.00	0.14 ± 0.00		

This table presents the results of spatiotemporal analyses of videofluoroscopic examination of swallowing and histological analyses, indicating the pre-and post-surgery values of the pharyngeal, esophageal areas, and the pharyngeal transit duration in different experimental groups. The experimental groups include those with bilateral and unilateral sectioning of the pharyngeal branch of the vagus nerve (bilateral and unilateral section groups), a sham-operated group (sham group), and a control group. The mean thickness of the thyropharyngeal (TP) and cricopharyngeal (CP) muscles for all groups is also noted. The data for the control group are shown in accordance with the respective time course of the study.

*p < 0.05, **p < 0.01.

The esophageal areas for the bilateral section group did not show a significant difference after the surgery (One-way ANOVA) (Figure 3B and Table 1). Although denervation of unilateral Ph-X in the unilateral section group elicited a transient decrease in the esophageal area, the reduction of the areas reached a stable state 1 month after the surgery; statistical significance was not apparent compared among all groups tested (One-way ANOVA).

Videofluoroscopic examination of swallowing also showed significantly higher pharyngeal transit duration after the surgery for bilateral and unilateral section groups (One-way ANOVA, Tukey’s multiple comparisons test, *p* < 0.01, <0.05) (see Figure 3C and Table 1). In addition, the pharyngeal transit duration for bilateral section group was statistically higher compared with that for unilateral section group at 3 weeks after the surgery (One-way ANOVA, Tukey’s multiple comparisons test, *p* < 0.05).

The time course of changes in the pharyngeal area, the esophageal area, and the pharyngeal transit duration are almost identical (repeated measures one-way ANOVA), and these areas and duration are not significantly different from sham and control groups (*t*-test) (Figures 3A–C). Figure 3D represents scatter plotting showing relationships between the pharyngeal area and the pharyngeal transit duration for all animals tested. A good linear correlation existed between these parameters (simple linear regression, *p* < 0.0001).

**Figure 4:**
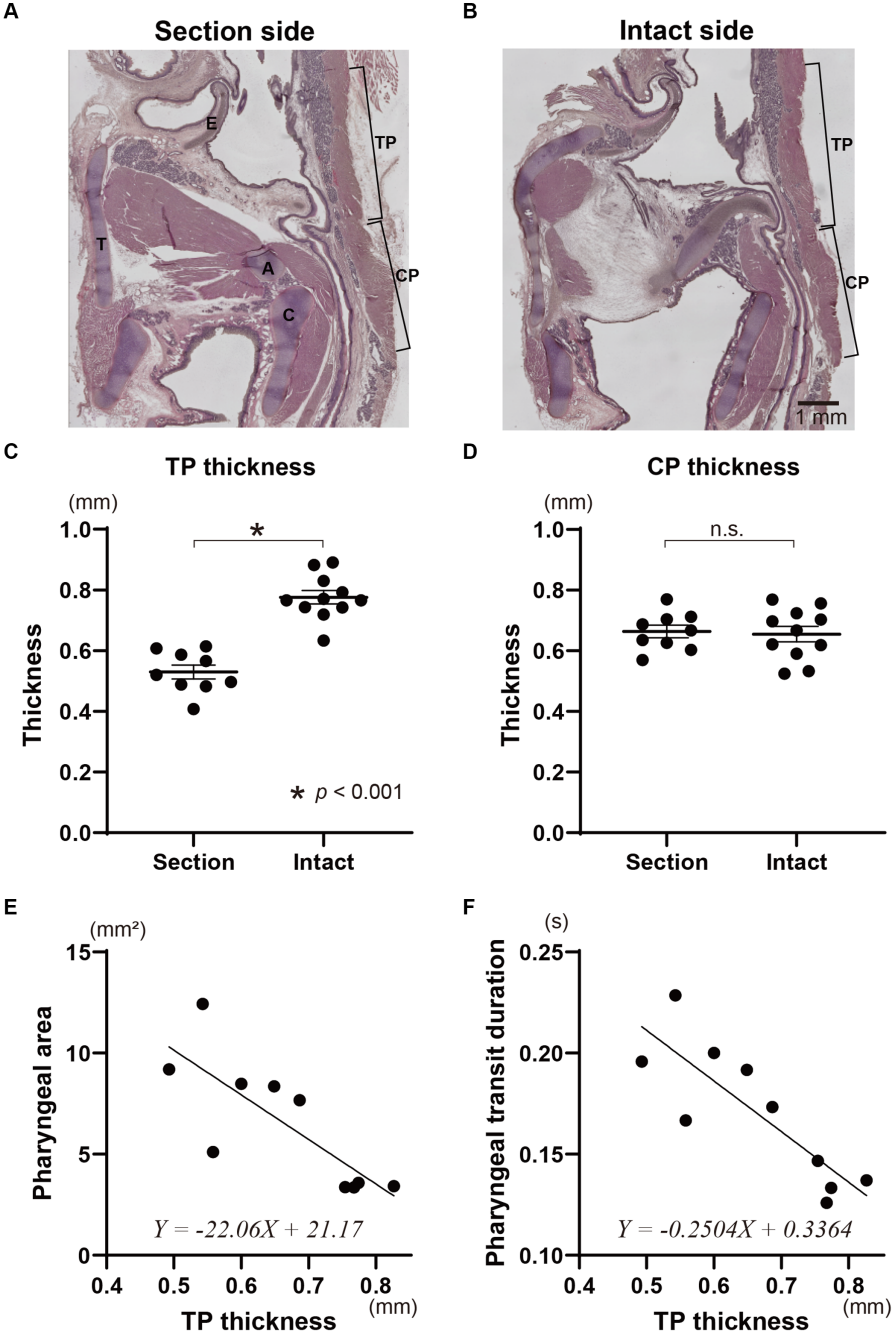
Histological evaluation of the pharyngeal constriction muscles due to the sectioning of the Ph-X and influence of the denervation on swallowing function. Representative sagittal tissue sections on the sectioned and intact sides of the pharynx in an animal with unilateral Ph-X section are shown in panels **(A,B)**, respectively. The thickness of the thyropharyngeal (TP) and cricopharyngeal (CP) muscles on the section and intact sides are represented in panels **(C,D)**. Relationships between pharyngeal area and the mean thickness of the TP muscle are shown in panel **(E)**. The pharyngeal transit duration and the thickness of the TP muscle for each animal are plotted in panel **(F)**. The linear regression lines and their formulae are provided in panels **(E,F)**. A, arytenoid cartilage; C, cricoid cartilage; E, epiglottis; T, thyroid cartilage.

The TP muscle on the sectioned side (0.53 ± 0.02 mm on average) was significantly thinner than that on the intact side (0.78 ± 0.02 mm on average) (*t*-test, *p* < 0.0001) (Figures 4A–C). In contrast, the thickness of the CP muscles on the sectioned and intact sides did not show a significant difference (0.66 ± 0.02 mm for the sectioned side, 0.65 ± 0.03 mm for the intact side) (*t*-test) (Figures 4A,B,D). The mean thickness of the bilateral TP muscles for each animal was significantly correlated with the pharyngeal area and pharyngeal transit duration using simple linear regression analysis (*p* < 0.01) (Figures 4E,F and Table 1). However, the mean thickness of the CP muscles showed no correlation to the pharyngeal area or pharyngeal transit duration.

## Discussion

The current study introduces a novel animal model that represents the dysfunction of pharyngeal contraction during pharyngeal swallowing. This model is produced by bilateral or unilateral transection of the Ph-X in guinea pigs; its characteristics were investigated using videofluoroscopy and histological verification. Data obtained suggest that denervation of the Ph-X could reduce the strength of pharyngeal contraction during pharyngeal swallowing possibly attributing to atrophy of the denervated TP muscles, resulting in a decrease in bolus speed.

The nerve transections were made alongside the lateral edge of the thyroid cartilage, resulting in a marked thinning of the TP muscle, and, thereby, attenuation of the pharyngeal constriction during swallowing (39–41). Meanwhile, the CP muscle functions as a sphincter muscle whose activity is inhibited in accordance with pharyngeal swallowing, determining the resistance of bolus passage at the esophageal entrance (38, 42–44). As such, the timing of the CP activity may influence bolus speed and pharyngeal residue during swallowing. The dysphagia model provided in this study could provide a suitable platform for pharyngeal dysphagia caused by diminished pharyngeal contraction forces.

Nevertheless, even with the transient decreases in bolus volume for each swallow after the injury, feeding behavior was maintained in all groups over time. The pharyngeal area and pharyngeal transit duration reached a stable level as their weight consistently increased 1 month after the surgery, probably allowing the support of swallowing movements from the glossopharyngeal nerve region to remain functional. Regarding histological and functional changes after nerve and muscle injury, denervation of the Ph-X is sufficient to cause changes in swallowing function 1 month after the surgery (18, 21, 31). The animal model created in this study therefore provides a non-lethal dysphagia model that can be used for pathological analysis and the development of new treatments for dysphagia caused by poor pharyngeal contraction.

The Ph-X nerve fibers mainly consist of motor efferent fibers projecting to striated muscles in the pharynx, including the TP and CP muscles (8, 41, 45–47). These fibers drive not only sequential contraction of the pharyngeal cavity during swallowing but also other respiratory and non-respiratory-related behaviors such as coughing (41, 47).

The motoneurons of the Ph-X are topographically distributed in the nucleus ambiguus (45). Previous studies reported that the membrane potentials of these neurons predominantly showed expiratory-related change during respiration (41, 46). These neurons also showed swallowing-related depolarization following transient hyperpolarization, which determines the timing of muscle contraction of pharyngeal constrictor muscles (47). Indeed, the expiratory-related and swallowing-related activities of the TP muscle were markedly decreased after the nerve transection.

Concerning the motor innervation in the pharyngeal region from the glossopharyngeal and vagal efferent through the pharyngeal plexus, massive damage of the pharyngeal plexus rostrocaudally along the pharynx may cause dysfunction of other swallowing-related muscles, such as the stylopharyngeal muscle controlled by the glossopharyngeal nerve (41, 45, 48). Further studies exploring the degeneration of the neuromuscular component caused by the Ph-X transaction may be necessary to investigate the detailed mechanisms of the muscle atrophy.

Although the nerve contains afferent fibers through which pharyngeal sensory signals are conveyed to the nucleus tractus solitarius, the superior laryngeal and glossopharyngeal nerves are significant afferent nerves that trigger pharyngeal swallowing (6, 26, 49–55). Thus, the influence of sensory denervation due to the transection of the Ph-X on swallowing function is likely to be minimal, which is in contrast to the dysphagia animal model produced by the transection of the superior laryngeal nerve.

In this animal model, pharyngeal contraction insufficiency induced the changes in the spatiotemporal parameters using videofluoroscopy. In this protocol for assessing swallowing function, fluid was directly infused into the oral cavity through a catheter to minimize the influence on oral movement, including licking and mastication. This testing procedure may facilitate evoking of sequential pharyngeal swallowing and may clarify the influence of the Ph-X section on pharyngeal swallowing. In the previous studies in the dysphagia animal models in rodents, swallowing function was assessed by videofluoroscopy using various parameters, such as inter-swallow intervals, swallow rate, pharyngeal and esophageal transit time, pharyngeal residue area, and bolus speed (18, 21, 23, 24, 28, 31, 56). In the present study, we focused on the evaluation of the motor performances caused by nerve denervation-induced pharyngeal constrictor muscle atrophy and the development of a suitable model for pharyngeal muscle incompetence. Therefore, initiation timing and likelihood of swallowing triggered by oropharyngeal bolus transition was not assessed. Additional behavioral testing for self-feeding circulation pattern to examine compensatory orofacial movement against feeding and swallowing difficulties will be necessary.

The study has limitations, including difficulty detecting the difference in the two-dimensional analysis of the pharyngeal passage area, particularly in the unilateral amputation model. However, the spatiotemporal and anatomical measures difference between animal groups of the Ph-X section and Sham-operated/control groups imply that swallowing dysfunction in the cranial motor denervation model could be appropriately evaluated by videofluoroscopic analyses. Although data obtained in this study provided significant information regarding swallowing function caused by Ph-X section, comparative studies with a larger sample size may be better powered to minimize the margin of error to confirm the efficacy of a new treatment for injured animals. Furthermore, additional studies are required including quantitative analyses of the electromyographic activities and immunohistochemical analyses for swallowing-related muscles before and at 1 month after the surgery to further evaluate functional and histological alteration after the denervation.

### Perspectives and conclusion

In this study, we developed the animal model that produced reduced pharyngeal contraction during swallowing by unilateral or bilateral transection of the Ph-X and assessed swallowing function using videofluoroscopy. This experimental model may provide essential information for the development of treatments for pharyngeal dysphagia and mechanisms related to the recovery process after injury, reinnervation, and nerve regeneration by swallowing impairment possibly caused by medullary stroke, neuromuscular disease, and surgical damage from head and neck cancer. This model organism also holds excellent promise for further research, as it could lead to significant advancements in diagnosing and treating swallowing disorders.

## Data availability statement

The raw data supporting the conclusions of this article will be made available by the authors, without undue reservation.

## Ethics statement

The animal study was approved by the local Universal Committee for the Use of Animals in Research (M2022-314). The study was conducted in accordance with the local legislation and institutional requirements.

## Author contributions

KH: Data curation, Formal analysis, Investigation, Methodology, Project administration, Resources, Writing – original draft, Funding acquisition. YS: Conceptualization, Data curation, Formal analysis, Funding acquisition, Investigation, Methodology, Project administration, Resources, Software, Supervision, Validation, Visualization, Writing – original draft, Writing – review & editing. MK: Formal analysis, Investigation, Project administration, Writing – review & editing, Funding acquisition. SK: Investigation, Writing – review & editing. RY: Investigation, Writing – review & editing. TI: Formal analysis, Writing – review & editing. TU: Conceptualization, Methodology, Supervision, Validation, Writing – review & editing. SH: Conceptualization, Methodology, Supervision, Validation, Writing – review & editing.
